# The state of library publishing in the health sciences in the United States and Canada

**DOI:** 10.5195/jmla.2026.2278

**Published:** 2026-10-01

**Authors:** Gina Genova

**Affiliations:** 1 gcgeno01@louisville.edu, Clinical Librarian, Kornhauser Health Sciences Library, University of Louisville, Louisville, KY

**Keywords:** Library publishing, scholarly communication

## Abstract

**Objective:**

Publishing initiatives led by academic libraries have grown over the past decade. They are often involved in the development of innovative publishing models and open initiatives, and a number of library publishers have worked in the health sciences. This study aims to assess the current state of library publishing in the health sciences (HS).

**Methods:**

The Library Publishing Directory survey and Library Publishing Coalition's member directory were accessed to identify active library publishing programs in the U.S. and Canada. Survey responses on disciplinary specialties and top publications were assessed, and individual publisher websites were accessed to inventory HS titles. Journals were checked for whether they were actively publishing and indexed.

**Results:**

One hundred eighteen survey respondents and 24 additional LPC members were assessed. Twenty-five percent of respondents named an HS subject as a disciplinary specialty, and 20% named an HS title as a top publication. Two hundred seventy-five individual HS titles from 81 different library publishers were identified. Fifty-three percent were journals, 39% open education resources, and 8% monographs. Medicine was the most common field for publications. Seventy-three percent of journals were actively publishing. Of those, 42% were indexed in DOAJ or a major HS database, and just over 10% appeared in PubMed or Scopus.

**Conclusions:**

Library publishers have a track record of working with a wide variety of HS researchers. Indexing of library-published journals is an area for improvement. HS librarians may be able to collaborate with their institutions' publishing programs to further develop these services and build relationships with researchers.

## INTRODUCTION

Scholarly publishing in the health sciences is an enormous enterprise, with over 1.5 million journal articles indexed just in PubMed in FY2023[1]. As with other disciplines, scholarly publishing in the health sciences is dominated by five commercial publishers: Elsevier, Sage, Springer Nature, Taylor & Francis, and Wiley, which combined publish a majority of journals indexed in Web of Science’s SCI-Expanded, SSCI, and AHCI [2]. The operation of these commercial journals is guided by a combination of publisher policies and recommendations from professional organizations, which include the International Committee of Medical Journal Editors and World Association of Medical Editors, and the discipline-agnostic Committee on Publication Ethics. These bodies provide best practices on issues ranging from criteria for authorship and authorship disputes, disclosure of conflicts of interest, peer review, corrections and retractions, the use of artificial intelligence, and more[3–6].

Researchers in the health sciences commonly face a pressure to “publish or perish,” with their opportunities for jobs, promotion or tenure, and funding often contingent on their ability to produce publications, particularly peer-reviewed journal articles [7]. Several factors may influence researchers’ choice of publishing outlet, including journal reputation and audience, personal experience with titles, the advice of colleagues or mentors, and the probability of acceptance for their article[8, 9]. Interestingly, researchers do not necessarily most frequently publish in journals that they regularly read, since readership habits skew more towards prestigious titles [10].

Recent trends in health sciences publishing mirror those in broader scholarly publishing. For example, the “gold” model of open access (OA) publication increased from 3% of scholarly publications in 2003 to 30% in 2022, in part due to requirements from funders like the National Institutes of Health [11, 12]. However, health sciences researchers’ knowledge of OA remains variable [9]. A related concern in health sciences publishing in recent years has been predatory or questionable journals, which are journals that exploit the OA model, namely the collection of article processing charges, but do not provide appropriate peer review processes and editorial services[9, 13, 14].

Over the past decade, academic libraries have become increasingly involved in scholarly communications services, including launching publishing programs of their own. Defined by the Library Publishing Coalition (LPC) as “the set of activities led by academic and research libraries and library consortia to support the creation, dissemination, and curation of scholarly, creative, and/or educational works,” library publishing offers an opportunity for libraries to establish themselves as leaders in scholarly communication and to support the development of open access and other innovative publishing models [15].

As early as 2008, 43% of ARL member libraries offered some type of publishing services, with another 21% of members planning to develop them. At that point, most library-published content was peer-reviewed, and library publishers aimed to develop new publishing models, not to duplicate traditional ones[16]. The LPC’s first *Library Publishing Directory* was published in 2014, which listed 116 individual publishers from five countries; the most recent version published in 2024 spotlighted 179 library publishers from 18 countries [15].

The scope of library publishers’ services vary widely, from that of a basic platform provider to much more in-depth publication services, such as obtaining DOIs or ISBNs, assistance with indexing in databases, copyright advisory, and technical production support[17–21]. As of 2024, a majority of library publishers offered DOI assignment, copyright advisory, metadata, training, ISSN registration, and non-copyright author advisory services[15].

### The Role of Library Publishers

As service-driven rather than commercial ventures, library publishers are well positioned to play a unique role in the scholarly communication ecosystem. Common motivations and priorities for library publishing programs include promotion of open access, offering less restrictive licenses, collaboration across campus, and providing services tailored to campus communities[22, 23]. Open access in particular is a key value, with most publications produced by library publishers available open access [23–27].

Library publishers are often able to provide an avenue for research that is in emerging fields, produced by small scholarly societies, or that may otherwise be too niche or otherwise overlooked by traditional commercial publishers[24, 28–30]. This can include research on issues relevant to marginalized or underserved communities, whom are sometimes neglected by larger commercial publishers [31].

Library publishers also often provide services to students. One common activity is the publication of undergraduate and graduate student journals, often with instructional programming to educate students about scholarly communications issues [24, 32–35]. Additionally, some library publishers produce open educational resources (OERs), which can help alleviate the burden of high textbook costs for students [21, 36–38]. Finally, library publishers provide a visible home for gray literature, such as conference proceedings, datasets, and dissertations/theses [22].

### Challenges in Library Publishing

Library publishing is not without challenges. Costs and ongoing funding are major concerns, as are the related issues of the sustainability and scalability of services given available staffing and other resources [16, 19, 21, 22, 28, 35, 39–41]. Another major concern is the training and professional development of librarians to manage publishing-related activities [22, 35]. As Schlosser points out, many library publishing programs are “bootstrapped” with minimal resources and support and as such may not meet best practices in scholarly publishing [26].

Library publishing also lacks standardization in workflows and procedures [39]. Some of this is unavoidable due to the different service levels provided by library publishers, the numerous platforms utilized by them, and the reality that different publication types, such as open educational resources, require different processes than journals or scholarly monographs [21]. Managing relationships with journal editors and navigating roles and expectations is also a variable process given the level of service provided by the library publisher, the developmental stage of journals, disciplinary norms, and the continuity (or lack thereof) within editorial boards [24, 32, 42, 43].

Another issue is establishing credibility for library publishers and obtaining the acceptance of library-published journals and monographs in research assessments [35]. While indexing in the Directory of Open Access Journals or Directory of Open Access Books is common for library-published titles, indexing in services like Scopus is less so, which can deter scholars operating under a “publish or perish” paradigm from choosing less-established venues for their work [28].

### Research Aims

Despite the number of library publishing ventures and the existence of partnerships between library publishers and health sciences researchers, there has been little discussion of library publishing within health sciences librarianship. This article aims to provide an assessment of the current state of library publishing efforts in the health sciences. How often are the health sciences a disciplinary priority for library publishers? How many health sciences titles have library publishers produced, and what types of publications are they involved in?

## METHODS

Publicly available data from the 2024 *Library Publishing Directory* survey (available at https://librarypublishing.org/lp-directory/) served as a key data source for this project, with several additional publishers identified using the Library Publishing Coalition’s (LPC) member list [15, 44]. The Library Publishing Coalition is the main professional organization for library publishers, and their *Directory* of publishing programs provides a detailed annual snapshot of activity in the field. Publishers do not have to be members of the LPC to participate in the survey and be included in the *Directory*, so the survey included a mix of member and non-member programs. English-language responses from publishers based in the U.S. or Canada were considered to avoid language barriers.

Survey respondents had the option to list up to five “disciplinary specialties” and “top publications” for their programs. Disciplinary specialties and top publications in any health sciences field were recorded. It should be noted that the *Library Publishing Directory* survey does not define a “disciplinary specialty” or “top publication,” so these designations may refer to number of publications in a discipline, disciplines with strong faculty-publisher relationships, the impact of publications, or other measures. “Health sciences” was considered broadly to encompass medicine, allied health fields, public health, medical humanities, clinical biomedical sciences, and so on. As such, life sciences or psychology titles with a clinical focus (e.g., mental health treatment, drug development) or for a health sciences audience (e.g., pre-medical students) were eligible for inclusion, but basic life sciences or general psychology titles were not.

Next, an inventory of health sciences titles published by library publishing programs was undertaken. Individual publisher websites, institutional repositories, and individual platforms (e.g., Open Journal Systems, Manifold) were accessed and their catalogs manually browsed for health sciences-related titles. Specific platforms used to publish journals or books were identified from survey responses about platform use, then located by searching Google with the key “institution name platform.” Not all publisher websites provided listings of their publications or links to catalogs, so additional checks of institutional repositories and specific platforms were deemed necessary.

The title, publisher, publication type, and health sciences field of journals, monographs, and complete open education resources (textbooks or full courses) in any field of the health sciences were recorded. Digitized archival material, reissues of historical university press publications, rehosts of other institutions’ open access publications, multimedia projects, dissertations or theses, conference proceedings, previously published institutional repository deposits, and more “one-off” OERs such as syllabi or individual case scenarios were excluded to focus on publication types where in-depth publisher support was most likely. Titles with multiple editions or translations were only counted once.

Additional data was recorded for journal titles. Their websites were accessed to determine their open access status and if the journals were active, which they were considered to be if they had published at least once in 2024 or 2025. Notes about several journal characteristics were also gathered from active journals’ websites: article processing charge, peer review, editor affiliation (internal vs external to the institution), student focus (run by students and/or dedicated to publishing student research), and subject coverage. Active journals’ ISSN registration and indexing status in Scopus, Web of Science, the Directory of Open Access Journals, MEDLINE, PubMed Central, CINAHL, and PsycINFO was assessed with the ISSN Portal, DOAJ web search, National Library of Medicine catalog, and database source lists [45–51].

The Carnegie Classification of U.S. institutions that published in the health sciences were recorded, and both U.S. and Canadian institutions that published in the health sciences were checked against lists of accredited programs in medicine, dentistry, veterinary medicine, optometry, and pharmacy [52–61].

Searches were initially done in March 2025 and updated in June 2025. All data was recorded in Excel.

Lastly, *Library Publishing Directory* data from 2014 (the first available) and 2019 were accessed, and data on programs’ top publications (2014 and 2019) and subject specialties (2019 only) were compared with current trends.

## RESULTS

### Survey Respondents

One hundred forty-two library publishers based in the United States or Canada were assessed for current library publishing activity. This included 118 respondents to the 2024 *Directory* survey and 24 additional Library Publishing Coalition members who had not submitted an updated entry for the most recent *Directory*. One hundred thirty (92%) of these publishers were academic libraries, and 10 (7%) were library consortia.

Of the 142 library publishers, a majority (86 / 61%) had a medical, dental, pharmacy, veterinary, and/or optometry school based at their institution or a member institution (in the case of consortia). Slightly over half (73 / 51%) specifically had a medical school at their institution or a member institution. Additionally, one program was based at an independent medical school, the University of Massachusetts Chan Medical School.

### Disciplinary Specialties and Top Publications

Of 118 evaluated 2024 survey respondents, 38 (32%) indicated some level of health sciences focus through a disciplinary specialty, top publication, or both. Fifteen (13%) named both a disciplinary specialty and a top publication in the health sciences, 14 (12%) named one of more health sciences disciplines as a disciplinary specialty but did not highlight specific publications, and nine (8%) listed a health sciences title as a top publication but did not describe themselves as specializing in the health sciences.

Of the 29 publishers (25%) who named one or more health sciences subjects as a disciplinary specialty, medicine or a medical specialty was most commonly named specialty, followed by general health sciences and public health (Table 1).

**Table 1:** 2024 Library Publishing Survey Responses Related to Health Sciences

Health Sciences Discipline	Publisher Specialty	Top Publications
Medicine (or medical specialty)	9	6
Health Sciences (general or unspecified)	6	2
Public Health	6	5
Nursing	5	5
Medical Humanities	3	5
Kinesiology/Sports Science	2	1
Biomedical Science	1	-
Health Science Education	1	-
Optometry	1	1
Allied Health Professions	-	4
Mental Health Professions	-	2
Consumer Health	-	1

Additionally, 24 publishers (20%) named one or more health sciences publications as a top publication. These publications were most often in medicine or a medical specialty, medical humanities, nursing, or public health (Table 1). Clinical specialties of top publications in medicine included lymphology, neurology, obstetrics/gynecology, and radiology. Of the 32 individual health sciences publications identified as a top publication, over two-thirds (22 / 69%) were journals, and most of the remainder (8 / 25%) were open education resources (OERs). One monograph and one dissertation in the health sciences were named as top publications.

Of the 86 survey respondents that had at least one health professional school based at their institution or a member institution, 29 (34%) named a health sciences discipline as a program specialty and/or a health sciences publication as a top publication.

Of the 32 respondents without an institutional health professional school, nine (28%) indicated some level of health sciences focus through disciplinary specialization, a top publication, or both. These publishers named specialties in health sciences fields that are not typically based at professional schools, such as nursing, medical humanities, kinesiology, and occupational therapy.

When publisher-identified disciplinary specialties and top publications are considered across time, library publisher involvement in the health sciences has been relatively consistent over the past decade. Roughly one-fifth of respondents named roughly 30 health sciences titles as a “top publication” in the 2014, 2019, and 2024 *Library Publishing Directory* surveys.

Similarly to 2024, medicine was the most-identified specialty for top publications in 2014 and 2019, but medical titles never comprised more than one-third of named top publications. Other fields represented in 2014’s top health sciences publications included biomedical sciences, general health sciences, health policy or law, kinesiology, medical humanities, mental health professions, nursing, pharmacy, and public health. In 2019, audiology, biomedical sciences, consumer health, general health sciences, health policy or law, health sciences librarianship, medical humanities, mental health professions, pharmacy, public health, and veterinary medicine were also represented among top publications.

However, the publication formats of publisher-identified top publications in the health sciences have shifted over time. Although journals have consistently been the most common format for top publications, they have become more common over time, and other publication types have changed. In 2014, as many theses or dissertations were called top publications as monographs. Additionally, in 2019, while journals and monographs were the most common top publication types, conference proceedings, dissertations or theses, exhibitions, newsletters, and OERs also all appeared once or twice. By 2024, journals and OERs were the most common top publication types.

### Publication Inventory

Two hundred seventy-five unique titles in the health sciences were identified from 81 individual library publishers’ websites, institutional repositories, and publishing platforms. A description of publisher demographics can be found in Table 2.

**Table 2:** Institutions Involved in Library Publishing in the Health Sciences

	Publishers with HS Titles (%) (N = 81)	Publishers With HS Focus* (%) (N = 38)
Country		
United States	67 (83)	33 (87)
Canada	14 (17)	5 (13)
Health Professional School		
Yes	56 (69)	29 (76)
No	25 (31)	9 (24)
Carnegie Classification		
R1	44 (54)	23 (61)
R2	7 (9)	3 (8)
Special Focus-Health Sciences	1 (1)	1 (3)
Special Focus-Arts and Sciences	2 (2)	–
Doctoral	1 (1)	1 (3)
Masters	2 (2)	2 (5)
Associate	1 (1)	–
Canadian, Health Prof. School	13 (16)	6 (16)
Canadian, No Health Prof. School	3 (4)	1 (3)
Consortium or University System	7 (9)	1 (3)

* Survey respondents that listed a health sciences field as a “disciplinary specialty” and/or a health sciences title as a “top publication.”

Publishers that have published in the health sciences published anywhere from 1 to 17 health sciences titles. Most publishers (42 / 52%) only published one or two health sciences titles, while a handful (4 / 5%) published ten or more health sciences titles. However, how prominent health sciences titles were in publishers’ catalogs varied somewhat by publisher size. Some programs with only a handful of available titles were heavily focused on health sciences (e.g., University of Massachusetts Chan Medical School), while some large, established programs produced larger numbers of health sciences titles but did not have a particular health sciences focus (e.g., the University of California System) [62, 63].

A slight majority (145 / 52%) of titles were journals. Most of the others (107 / 39%) were OERs, and a small number (23 / 8%) were monographs. Monographs included a mix of consumer-oriented titles, such as memoirs; scholarly monographs on topics like history, policy, and social issues; and clinical manuals.

Across all publications, over a dozen health sciences disciplines were represented. Medicine (59 / 22%) was the most-represented discipline, followed by general health sciences, biomedical sciences, nursing, and public health (Table 3).

**Table 3:** Health Sciences Disciplines of Library-Published Titles

Discipline	Publications (%) N = 275*	Journals (%) N = 145	OERs (%) N = 107
Medicine (or medical specialty)	59 (22)	43 (30)	12 (11)
Health Sciences (general or unspecified)	38 (14)	25 (17)	11 (10)
Biomedical Sciences	27 (10)	5 (3)	22 (21)
Nursing	27 (10)	6 (4)	21 (20)
Public Health	23 (8)	16 (11)	7 (7)
Mental Health Professions	21 (8)	13 (9)	8 (7)
Medical Humanities	18 (7)	12 (8)	-
Veterinary Medicine	14 (5)	1 (1)	11 (10)
Nutrition/Dietetics	9 (3)	3 (2)	6 (6)
Kinesiology/Sports Science	8 (3)	5 (3)	3 (3)
Health Sciences Librarianship	7 (3)	3 (2)	-
Health Policy/Law	6 (2)	4 (3)	1 (1)
Consumer Health	5 (2)	-	-
Pharmacy	4 (1)	1 (1)	3 (3)
Dentistry	3 (1)	2 (1)	1 (1)
Speech-Language Pathology/Audiology	2 (1)	1 (1)	1 (1)
Complementary/Alternative Medicine	1 (<1)	-	-
Occupational Therapy	1 (<1)	1 (1)	-
Optometry	1 (<1)	1 (1)	-
Physical Therapy	1 (<1)	1 (1)	-

* including 23 monographs.

Among the 145 journals, medicine was more of a dominant discipline (43 / 30%), although it still comprised less than a third of total publications. General health sciences, public health, mental health professions, and the medical humanities were also prominent in this subset of titles (Table 3). Of note, several of the represented mental health professions were smaller fields, such as financial therapy, music therapy, and sand therapy.

Nearly all journals (143 / 99%) were open access. One hundred six (73%) journals were identified as active, including 28 (85% of) student-run journals. Additional notable journal characteristics are highlighted in Table 4.

**Table 4:** Selected Journal Characteristics

Trait	Journals (n = 145) (%)	Fields
Student-Run/Student Research	33 (23)	Medicine (14), General Health Sciences (10), Public Health (2), Biomedical Sciences (1), Health Law/Policy (1), Kinesiology/Sports Science (1), Medical Humanities (1), Nursing (1)
Health Professions Education	10 (7)	Allied Health (3), Medicine (2), General Health Sciences (2), Nursing (2), Mental Health Professions (1)
Local/Regional Focus or State Scholarly Society (excl. student journals)	9 (6)	Public Health (7), Medicine (1), Mental Health Professions (1)
Global Health	7 (5)	General Health Sciences (5), Medicine (1), Mental Health Professions (1)

* Journals on “global health” broadly were classified as general health sciences since most appear to publish in medicine and public health broadly.

Among OERs, biomedical sciences (22 / 21%) and nursing (21 / 20%) were the most common fields, followed by medicine, general health sciences, and veterinary medicine. Most of the biomedical sciences OERs (15 / 68%) were anatomy/physiology textbooks aimed at health professional students. Overall, fewer fields had OERs published by libraries than journals (Table 3).

### Indexing & Operations of Active Journals

Of the 145 identified journal titles that had ever published, 106 (73%) published at least once in either 2024 or 2025 and were therefore considered to be active. Eighty-five (80%) of active journal titles had an identifiable ISSN. One hundred four (98%) active journals were open access, of which most (60 / 58%) could be confirmed not to charge article processing fees. Additionally, the vast majority were peer reviewed (92 / 87%), with double-blind review being the most commonly utilized (47 / 44%). Journals that were not peer-reviewed were often student-focused and employed an editorial review process (Table 5).

**Table 5:** Operational Characteristics of Active Library-Published Health Sciences Journals

Trait	Journals (n = 106) (%)	Non-Student Journals (n = 78) (%)
Article Processing Charge		
Yes	7 (7)	7 (9)
No	60 (58)	51 (65)
N/A (non-OA)	2 (2)	2 (3)
Unclear	37 (35)	18 (23)
Peer Review		
Blinded (details unclear)	11 (10)	8 (10)
Single-Blind	11 (10)	10 (13)
Double-Blind	47 (44)	37 (47)
Optional Double-Blind/Open	2 (2)	2 (3)
Open	1 (1)	1 (1)
Unspecified Peer Review	20 (19)	17 (22)
Any Peer Review	92 (87)	75 (96)
Not Peer Reviewed	6 (6)	0
Unclear	8 (8)	3 (4)
Editor Affiliation		
Internal	65 (61)	38 (49)
External	37 (35)	36 (47)
Unclear	4 (4)	4 (5)
Society/Organization Affiliation		
Yes	24 (23)	24 (31)
No	82 (77)	54 (69)

Only 43 (42%) active journals were indexed in at least one of the sources inspected. The Directory of Open Access Journals (DOAJ) was by far the most common place journals were indexed (33 / 31% of all journals, 32% of OA journals). Only eleven journals (10%) were indexed in PubMed, and only five (5%) were indexed in Web of Science and thus had an impact factor (Table 6). The most-indexed journals were the *Journal of the Medical Library Association* hosted by Pitt Open Library Publishing and the *Western Journal of Emergency Medicine* hosted by eScholarship University of California, which were both indexed in all evaluated sources but PsycINFO.

**Table 6:** Indexing of Active Library-Published Health Sciences Journals

Source	Journals (n = 106) (%)	Non-Student Journals (n = 78) (%)
CINAHL	15 (14)	13 (17)
DOAJ	33 (31)	29 (37)
PsycINFO	4 (3)	4 (5)
PubMed	11 (10)	10 (13)
*MEDLINE*	*6 (6)*	*5 (6)*
*PubMed Central Only*	*5 (5)*	*5 (6)*
Scopus	11 (10)	10 (13)
Web of Science	5 (5)	5 (6)
*Emerging Sources Citation Index (ESCI)*	*2 (2)*	*2 (3)*
*Science Citation Index Expanded (SCI-E)*	*2 (2)*	*2 (3)*
*Social Sciences Citation Index (SSCI)*	*1 (1)*	*1 (1)*

## DISCUSSION

Most existing library publishers are based at institutions with at least one health professional school, and thus many health sciences librarians already work at institutions with an active library publishing program. This presents several opportunities for collaboration between library publishing colleagues and health sciences researchers interested in publishing.

There are some promising signs as to the strength of library publishing ventures in the health sciences. For example, nearly all non-student journals are peer-reviewed, and nearly a third of non-student journals are affiliated with an external professional organization or scholarly society. However, only one third of library publishers with a health professional school based at their institution identify the health sciences as a disciplinary specialty or area of top publication, which suggests room for growth in building relationships between academic health sciences librarians and their patrons and library publishers.

Responses to the *Library Publishing Directory* survey indicate that library publishers’ involvement in the health sciences has been relatively consistent over the past decade. However, the particulars of their involvement appear to have shifted. Earlier survey responses indicated that publishers’ work in the health sciences was frequently with grey literature like dissertations/theses. Currently, programs highlight more formal health sciences publications like journals and open educational resources (OERs) when asked to name “top publications.”

One area of frequent involvement for library publishers is student research. One-fifth of identified journal titles were student-focused, of which the vast majority are active. Related to this, journals dedicated to health professions education research are somewhat common from library publishers, with ten titles (nine active) published in multiple fields. These two journal trends, combined with the growing prominence in health sciences OER publications in recent years, highlight the role of library publishers in advancing health professions education.

Library publishers also seem to have developed a niche in place-specific public health research. This is seen in titles like *Journal of Appalachian Health* from the University of Kentucky and the publication of their state public health association’s journal by library publishers at Georgia Southern University, the Ohio State University, Grand Valley State University in Michigan, and James Madison University in Virginia. This makes sense—if local public health research is considered too niche for mainstream journals, or if local- and state-level public health associations are hesitant or unable to contract with a major commercial publisher to produce their journal, partnering with library in the area can ensure that relevant research is still made accessible to the community.

Related to local interests, library publishers are sometimes offered as promising partners for small or emerging fields, and this has been observed to some extent in the health sciences [29]. Library publishers have published journals on topics such as graphic medicine, financial therapy, Muslim mental health, music therapy education, optometry history, sand therapy, and veterinary forensic sciences. These highly specialized titles indicate that library publishers can be viable partners for these types of projects.

It is notable that library publisher involvement in the health sciences spans multiple disciplines. Although medicine is the most common field for health sciences titles produced by library publishers, it accounts for less than one quarter of their total health sciences publications. This suggests that working with library publishers need not be limited to health sciences librarians in medical school settings, but can also include librarians working with nursing, public health, or allied health faculty and students at less research-intensive institutions.

However, there may be challenges in expanding library publisher involvement in the health sciences. Library publishing programs frequently grapple with issues related to costs, sustainability, and the scaling of services [16, 19, 22, 28, 39, 40]. Although there are no health sciences-specific reports of these challenges, it can be expected that similar issues will be encountered while developing library publisher partnerships with health sciences researchers. Another challenge that is not specific to the health sciences but may be especially salient for health sciences researchers is the indexing status and impact factors of library-published journals [28]. Many health sciences faculty are evaluated not only on the amount of research they publish, but *where* they publish it, and indexes like Scopus and metrics like impact factor are frequently used, however problematically, as proxies for outlet quality.

However, library-published health sciences journals are infrequently indexed, despite best practices like peer review being the norm. Part of this is likely because many library-published journals are relatively new and have not established the track record required for indexing. However, there is at least one example of a library-published journal being rejected by an indexing service, partly due to reviewers’ misunderstanding of the journal’s relationship to the library and the publishing platform used [64]. Helping faculty editors understand indexing requirements and assisting with the indexing process could be a valuable role for health sciences librarians and library publishers interested in growing their services to the health sciences researchers.

Overall, it is clear that health sciences researchers across disciplines work with library publishers with some frequency, both at institutions with health sciences professional schools and those without. Although the resources and staff needed to implement a publishing program de novo is probably beyond the reach of most individual health sciences libraries, health sciences librarians could collaborate with non-health sciences or consortial partners to further library publishing work. This could include raising awareness of library publishing outlets among health sciences researchers; connecting would-be journal editors or OER authors to publishing services, especially in niche or local-interest fields; and providing guidance to journal editors on issues salient to health sciences researchers, such as indexing.

### Limitations

One limitation of this study is the sample selection. This study relied on the *Library Publishing Directory* and the Library Publishing Coalition’s (LPC) member directory to identify active library publishing programs in the U.S. and Canada. Publishers do not have to be LPC members to be included in the *Directory*, so the sample is not restricted to LPC members, but there are likely some library publishers that are not LPC members and did not participate in the *Directory* survey and therefore were not evaluated. Additionally, while the decision to focus on English-language U.S. and Canadian publishers was made to avoid potential language barriers, library publishers do exist worldwide and in languages other than English, and library publisher work in the health sciences may differ in other countries. A more global survey utilizing additional data sources may surface additional trends.

Other limitations exist with the data sources. The *Library Publishing Directory* survey asks questions about publishing programs’ structure and staffing as a whole, so while some insights can be gleaned from those responses, they are not specific to publishers’ work with health sciences researchers. Given that health sciences publishing is a small portion of most publishers’ work, there may be additional opportunities, challenges, or other nuances to these publishing ventures that are not reflected in program-wide survey data.

Survey responses also don’t capture how involved library publishers are with any given title. Library publisher services range from simple platform provision to professionalized, full-service publication support, and publishers may provide different levels of services to different projects within their portfolios. As such, the details of exactly *how* library publishers support health sciences publications, and what barriers or opportunities exist for different support models, cannot be determined by the data available for this paper.

Finally, it cannot be guaranteed that all health sciences publications from the assessed publishers have been evaluated. Certain publication types were deliberately excluded due to this project’s scope, and it is possible that eligible publications were missed during the inventory. There is no standard website design for library publisher webpages and no standard organization for institutional repositories that host library-published content. The author visited websites multiple times and supplemented website links with Google searches to attempt to fill as many gaps as possible, but it is possible some omissions still occurred. Additionally, some journals that were identified as inactive for this study but were not officially “archived” may resume publishing in the future.

## CONCLUSION

Academic libraries and library consortia have growing involvement in the provision of publication services. As interest in open access publication and innovative publication models increases, library publishers offer an intriguing alternative to traditional commercial publishers. These publishers have a track record of working with health sciences researchers in multiple disciplines to disseminate scholarship and open educational resources, with notable involvement in areas like student research, local public health research, and health sciences education. Health sciences librarians have the opportunity to collaborate with their colleagues working in library publishing to raise awareness of these publishing opportunities and, potentially, to provide guidance on health sciences-relevant issues during project development, such as database indexing.

## Data Availability

*Library Publishing Directory* survey data is available at https://librarypublishing.org/lp-directory/. Data related to the inventoried health sciences publications are available in the Open Science Framework at https://osf.io/nxbqz/.
